# Designing Biomimetic Learning Environments for Animal Welfare Education: A Gamified Approach

**DOI:** 10.3390/biomimetics10110769

**Published:** 2025-11-13

**Authors:** Ebru Emsen, Bahadir Baran Odevci, Muzeyyen Kutluca Korkmaz, Fatma Alshamsi, Alyaziya Alkaabi

**Affiliations:** 1 Integrative Agriculture, College of Agriculture & Veterinary Medicine, United Arab Emirates University, Al Ain P.O. Box 15551, United Arab Emirates202303867@uaeu.ac.ae (A.A.); 2 Imona Technologies, ITU Ari Teknokent, 34467 Istanbul, Turkey; bahadir.odevci@gmail.com; 3 Department of Animal Science, Faculty of Agriculture, Malatya Turgut Ozal University, 44210 Malatya, Turkey; muzeyyen.korkmaz@ozal.edu.tr

**Keywords:** biomimicry, gamification, animal welfare education

## Abstract

Animal welfare education requires pedagogical models that bridge conceptual knowledge with practice. This study presents GamifyWELL, a biomimetic, gamified learning environment for students, farmers, and veterinary technicians. Grounded in ecological principles of adaptation, diversification, and niche specialization, the design emulates how living systems evolve through feedback and cooperation. These principles were translated into an instructional model that integrates a core pathway (Pre-Test, Levels 1–4, Post-Test) with optional enrichment tasks and a role-specific Reward Marketplace. Question formats are constant across levels (MCQ, image-based, video-based) while cognitive difficulty increases, culminating in Positive Welfare scenarios. We describe the learning design structure and report preliminary implementation observations using a mixed-methods evaluation plan (pre/post knowledge assessments and engagement indicators). Results from early deployment indicate strong usability and engagement, with high voluntary uptake of enrichment tasks and positive learner feedback on role-tailored rewards; full empirical testing is in progress. Findings support the feasibility and pedagogical promise of biomimetic gamification to enhance knowledge, motivation, and intended practice in animal welfare education. GamifyWELL offers a replicable framework for nature-inspired instructional design that can be extended to allied sustainability domains.

## 1. Introduction

Animal welfare is becoming increasingly important as sustainability goals become more critical and consumer awareness dramatically increases among a growing population. This current trend addresses the strong link between animal welfare and human well-being, now covered by the “One Health” concept, which indicates mutual dependency on a healthy living environment [1]. At this point, there is an urgent need for more innovative pedagogical approaches to animal welfare education, especially considering the increasing disconnections between humans and animals in industrialized lifestyles [2]. Developing sophisticated educational tools can effectively close the gap between theoretical knowledge and practical experience, enhancing the understanding of animal welfare.

Biomimicry, defined as borrowing ideas from nature, offers a powerful framework for designing immersive learning experiences that emulate natural systems and animal behaviors. Then, the interdisciplinary cooperation of biology and technology, characterized as biomimetics, can open a new door for innovative educational strategies that enhance empathy and ethical understanding towards animals [3]. In translating biomimetic theory into pedagogy, concepts such as adaptation and feedback loops informed the stepwise learning progression, while niche specialization guided the differentiation of learner roles and reward types. Thus, each instructional element was purposefully aligned with a biological process to ensure coherence between ecological metaphor and educational design.

This conceptual alignment draws upon constructivist learning theory, which posits that knowledge is actively constructed through interaction and reflection rather than passively received. Within this paradigm, biomimetic design serves as an epistemic metaphor—linking ecological adaptation and feedback processes with learner-centered knowledge building. By modeling learning pathways on ecological systems, the framework encourages self-regulation, adaptation, and mutual reinforcement among cognitive, emotional, and behavioral dimensions of welfare education. This integration establishes a theoretical bridge between biomimicry and constructivism, positioning GamifyWELL as both ecologically inspired and pedagogically grounded. This method extends beyond mere imitation, delving into the underlying principles that allow natural systems to thrive efficiently and sustainably, providing a rich source of inspiration for complex problem-solving [4]. This methodology inherently supports interdisciplinary learning by merging biological principles with practical animal management, fostering a comprehensive understanding of sustainable practices in livestock production and environmental quality [5]. By engaging learners actively with complex systems and real-world problems, these environments cultivate a sustainability-aligned mindset essential for addressing evolving environmental challenges [6]. Furthermore, the integration of digital tools, such as gamification and virtual reality, within these biomimetic frameworks can significantly enhance student engagement and knowledge retention, particularly in fostering pro-environmental behaviors and environmental identity [7]. This integration allows for the exploration of complex biological principles and ecological functions, providing a framework for sustainable development across various sectors [8]. GamifyWELL primarily employs a gamification approach—applying game elements such as levels, mastery thresholds, and rewards within a non-game educational context—while selectively integrating aspects of game-based learning to enhance engagement rather than functioning as a full serious-game simulation, clarifying that GamifyWELL is a gamified learning environment that applies game elements (levels, rewards, feedback) within a non-game educational context, and therefore represents a gamified design framework rather than a full game-based or serious-game simulation.

Digital platforms allow students to engage with sophisticated ecological models and management simulations, thereby developing the critical thinking and problem-solving abilities crucial for tackling contemporary animal welfare issues. Moreover, these immersive digital environments facilitate the application of theoretical knowledge to practical scenarios, allowing students to experiment with different welfare strategies and observe their simulated outcomes in a controlled, risk-free setting. By simulating realistic agricultural production principles within a classroom setting, extended reality can bridge the theory–practice gap, providing students with necessary hands-on experience without the geographical and resource constraints of traditional methods [9,10]. This would allow for repeated engagement with complex animal welfare dilemmas, fostering critical thinking and nuanced understanding without direct imposition on live animals. These interactive simulations can effectively train future agricultural professionals in identifying nuanced signs of animal distress and implementing preventative measures, thereby directly improving animal well-being on farms [11]. This not only optimizes training efficiency but also significantly reduces the ethical and logistical challenges associated with using live animals for educational purposes.

This study focused on creating gamification for training in small ruminant welfare, integrating biomimetic principles to improve learning and foster a deeper comprehension of animal behavior and needs. This approach builds on previous work, such as a prospective cohort study that used digital simulation of a pig farm to teach agricultural students how to reduce piglet mortality [12]. Specifically, this study leverages game-based learning, which provides active, experiential, and problem-based learning with immediate feedback, preparing students for practical application in animal handling and welfare management. We hypothesize that such an approach, combining biomimicry with gamification, will lead to enhanced cognitive and affective learning outcomes, thereby preparing students for real-world animal welfare challenges. This paper presents the conceptual design and pilot implementation of the GamifyWELL framework rather than a full-scale effectiveness trial. Quantitative and qualitative feedback were collected to inform the refinement of the module for future controlled studies.

## 2. Literature Review

### 2.1. Educational Instruments for Animal Welfare

Traditional educational instruments for animal welfare often include lectures, textbooks, and direct animal interaction, which, while foundational, frequently lack the immersive and interactive elements necessary for deep conceptual understanding and skill acquisition. However, these conventional methods often present challenges in replicating complex, real-world scenarios and providing immediate, personalized feedback, which are crucial for developing nuanced observational skills and ethical decision-making in animal welfare contexts [12]. The scarcity of practical training opportunities and hands-on clinical skill acquisition further compounds these limitations, particularly for veterinary students and farm workers [13]. Moreover, the logistical and ethical considerations associated with direct animal handling for educational purposes often limit the scope and frequency of such practical experiences [14]. This underscores the need for innovative educational tools that can bridge this gap by offering engaging, repeatable, and ethically sound alternatives for animal welfare education. This challenge is particularly pronounced in areas like lameness assessment, where novices struggle to reliably identify gait abnormalities without systematic practice and feedback [15]. Consequently, the efficacy of traditional instruction in animal welfare, particularly when it relies on didactic methods, has often been questioned due to its limited success in effectively conveying proper etiquette in animal handling and fostering a comprehensive understanding of ethical issues [14]. This limitation highlights the potential of digital learning technologies to serve as boundary objects, thereby transforming farmers’ perceptions of animal welfare and management practices [16]. Gamified design, which incorporates biophilic design, has demonstrated significant potential in fostering sustainable behaviors and enhancing environmental awareness by immersing users in virtual environments that reflect natural principles. This pedagogical approach not only makes learning more compelling and realistic through immersive virtual environments but also supports traditional teaching methodologies by actively engaging students in complex scenarios [12]. This is particularly relevant given the documented shift in educational paradigms, driven by student demands for improved access to learning materials, flexible study options, and enriched learning experiences [17].

### 2.2. Gamification in Animal Science

The integration of gamified elements into animal science education, particularly through virtual reality platforms, offers an innovative solution to these limitations by providing engaging and interactive learning experiences [14]. Following Deterding et al. [18] and Hamari et al. [19], we distinguish gamification (the use of game elements in non-game contexts) from game-based learning and gamification (complete game environments), positioning GamifyWELL within the former category. This approach leverages digital game-based learning to purposefully integrate technology, allowing educators to maintain focus on learning objectives while exploring innovative teaching methodologies [20]. Digital educational games, such as simulations of pig farms, have shown promise in improving learning outcomes, particularly for lower-performing students, by offering an additional, engaging training resource [12]. For instance, a study demonstrated that 60% of players rapidly acquired forelimb lameness detection skills in under 10 min when engaging with a gamification, correctly assessing 30% movement asymmetry [15]. This highlights the potential of gamification to facilitate rapid skill acquisition and improve diagnostic accuracy in complex areas of animal welfare [12].

This technological integration addresses the fundamental imperative of responsible animal use in research by offering a risk-free environment for practicing animal handling, thereby bridging the gap where traditional tertiary education often falls short. Moreover, the use of virtual reality in conjunction with gamification and didactic pedagogy offers a promising avenue for reinforcing practical knowledge and improving task performance in animal science training [14].

## 3. Materials and Methods

The study follows a design-based research approach focusing on the development and initial testing of the GamifyWELL modules. This section outlines the experimental design, participants, intervention details, data collection instruments, and analytical approaches employed to evaluate the effectiveness of biomimetic learning environments in animal science education. A small informal pilot (*n* = 10 undergraduate students) was conducted for initial usability feedback during a regular teaching session. The activity took place in a supervised computer-lab setting and lasted approximately 60 min. The methodology was structured to rigorously assess the impact of game-based nature models on welfare training, encompassing both quantitative measures of learning outcomes and qualitative evaluations of student engagement and perception. Engagement indicators included both behavioral and self-reported measures, such as task completion rate, frequency of voluntary participation in enrichment activities, average time-on-task, and system log-ins. Qualitative feedback was obtained through a short post-session questionnaire using 5-point Likert scales on motivation and perceived learning value. Cronbach’s α = 0.82 was calculated for internal consistency of the motivation scale used in this feedback instrument. The pre- and post-tests were reviewed by three subject-matter experts to ensure content validity.

### 3.1. App Design as a Biomimetic Learning Environment

#### 3.1.1. Instructional Design Framework

The gamified learning environment (GamifyWELL) was conceived as a biomimetic instructional tool to facilitate structured learning in animal welfare for diverse stakeholders, including undergraduate students, farmers, and veterinary technicians. The pedagogical design was anchored in the principle of biomimicry, wherein learning architectures emulate adaptive processes and feedback mechanisms characteristic of natural ecosystems.

#### 3.1.2. Core Pathway (Dependent Tasks)

The core learning pathway was operationalized through a sequential progression of interdependent tasks, reflecting the stepwise adaptation and complexity-building observed in biological systems. Each level was self-paced, allowing learners to progress after achieving an 80% mastery threshold. Optional hints and replay functions minimized cognitive overload and supported gradual adaptation for users new to digital learning. Each stage of the pathway corresponded to a distinct pedagogical function and simultaneously embodied a biomimetic principle of adaptation (Table 1).

**Figure 1:**
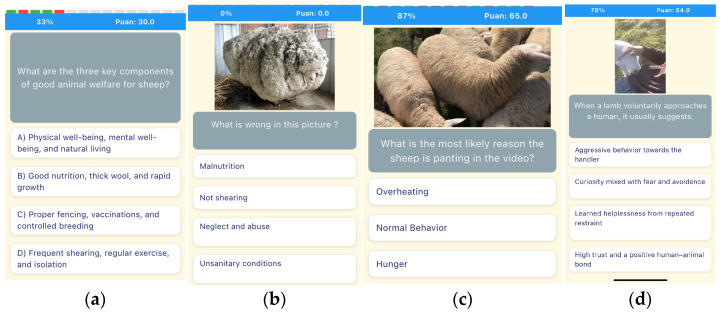
Example question formats from the GamifyWELL application: (**a**) Multiple-choice item from Level 1, illustrating basic awareness tasks; (**b**) Image-based recognition question from Level 2, designed for intermediate practice; (**c**) Video-based situational problem-solving item at Level 3, addressing advanced welfare issues; (**d**) Positive Welfare scenario from Level 4, focusing on enrichment and affiliative behaviors.

Figure 1 provides illustrative examples of the question formats embedded within the GamifyWELL application, including multiple-choice, image-based, and video-based tasks. These items were designed to increase in difficulty across Levels 1–4, culminating in advanced Positive Welfare scenarios.

This progression embodies a biomimetic adaptive cycle, wherein learners navigate progressively complex challenges and integrate new knowledge, closely paralleling the incremental adaptation and refinement processes observed in ecological systems.

#### 3.1.3. Enrichment Pathways (Independent Tasks)

To mirror the diversification and social learning dynamics inherent in natural ecosystems, participants were provided with optional enrichment tasks. These pathways extended learning beyond the linear progression of the core sequence and embodied principles of biodiversity, cooperation, and competition. Functionally, they served both motivational purposes (additional scoring and recognition) and pedagogical purposes (content enrichment through user-generated contributions). In this way, the game design ensured that knowledge construction was not static but continuously adaptive, echoing the evolutionary dynamics of ecological systems (Table 2).

These independent pathways contributed not only to reinforcement of motivation but also to the continuous evolution of the learning ecosystem. User-generated inputs were systematically incorporated into the question bank and scenario repository, thereby ensuring that the educational content remained dynamic, adaptive, and enriched by collective participation.

#### 3.1.4. Reward Marketplace (Reinforcement Hub)

All learning pathways converged in the Reward Marketplace, which functioned as a central reinforcement hub designed to sustain motivation and provide personalized recognition. Rewards were intentionally context-specific and role-sensitive, reflecting the ecological principle of niche specialization, where each actor within an ecosystem benefits in accordance with its functional role. In the gamified environment, this ensured that learners across stakeholder groups (students, farmers, and veterinary technicians) were rewarded in ways that resonated with their professional identity and learning context (Table 3).

This differentiation reflects not only ecological niche specialization but also pedagogical tailoring—students engage primarily with conceptual learning tasks, farmers with applied welfare practices, and technicians with diagnostic and facilitation competencies. This role-specific reward distribution system operationalizes the principle of niche differentiation, whereby individuals derive benefits congruent with their ecological or educational role. In doing so, the Reward Marketplace not only sustains engagement but also enhances system resilience, mirroring the adaptive stability of natural ecosystems.

### 3.2. Visualization of System Architecture

**Figure 2:**
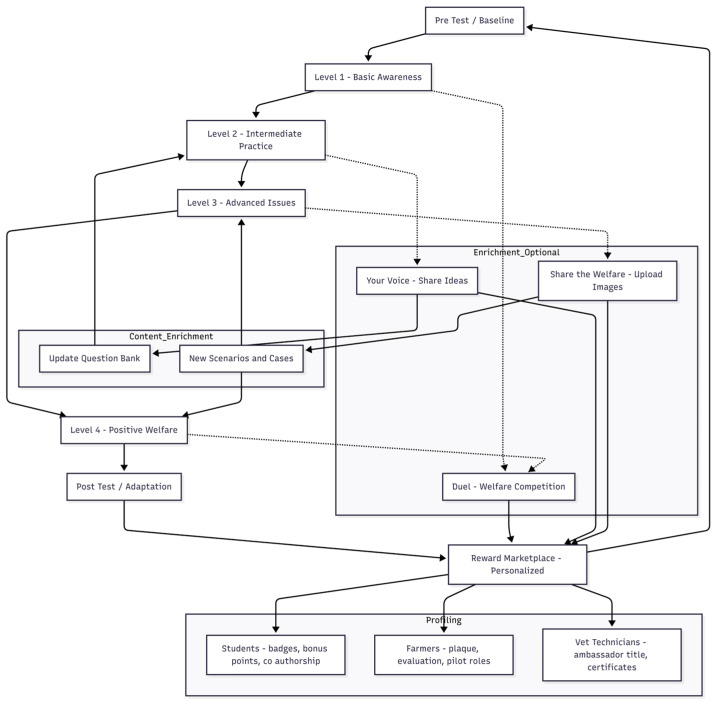
Biomimetic gamified learning architecture of GamifyWELL. Solid arrows indicate the sequential dependent pathway (Pre-Test → Levels 1–4 → Post-Test → Reward Marketplace), whereas dashed arrows denote independent enrichment tasks. The Reward Marketplace functions as a reinforcement hub, profiling rewards according to user role and sustaining the adaptive cycle.

The biomimetic architecture of the gamified learning environment is presented in Figure 2. Solid-line sequences denote the dependent (core) pathway, while dashed-line connections represent enrichment tasks. Feedback loops from enrichment tasks into the content enrichment module illustrate the adaptive, self-renewing character of the learning system.

## 4. Results

The development of GamifyWELL resulted in the successful implementation of a biomimetic gamified framework for animal welfare education. The system integrated three complementary components: a core learning pathway (sequential dependent tasks from pre-test to Level 4 and post-test), enrichment pathways (independent and socially driven challenges), and a Reward Marketplace (role-specific reinforcement hub). Collectively, these elements established a dynamic and adaptive environment that mirrors the feedback loops and specialization principles of natural ecosystems.

The core pathway ensured that learners progressed through increasingly complex tasks, moving from baseline knowledge assessment to mastery of Positive Welfare concepts. This sequential structure operationalized the principle of incremental adaptation, enabling learners to consolidate knowledge in stages and build resilience against cognitive overload. The enrichment tasks provided opportunities for voluntary participation beyond the linear pathway. Activities such as Welfare Duels, sharing ideas, and visual submissions intended to promote social learning, creativity, and peer-to-peer exchange. These contributions continuously enriched the content bank, ensuring that the learning ecosystem remained adaptive and self-renewing. The Reward Marketplace functioned as a reinforcement hub, offering differentiated incentives tailored to students, farmers, and veterinary technicians. By mirroring ecological niche specialization, the marketplace sustained engagement and strengthened user identification with the training outcomes.

Preliminary pilot observations indicated strong user engagement, high voluntary uptake of enrichment activities, and positive feedback on the relevance of role-specific rewards. Engagement and survey data were compared descriptively at an aggregated level to ensure anonymity, providing an overview of behavioral and self-reported indicators without linking individual responses. High voluntary uptake reflected behavioral participation, while positive learner feedback represented self-reported motivation and satisfaction. While systematic empirical testing is ongoing, these initial outcomes suggest that the biomimetic gamification design is likely to enhance knowledge retention, foster more positive attitudes toward animal welfare, and increase learners’ confidence in applying welfare principles in real-world contexts.

## 5. Discussion

This study demonstrates that the integration of biomimetic gamification into animal welfare training can effectively bridge theoretical understanding with practical application. By modeling learning processes on ecological principles such as adaptation and diversification, the GamifyWELL platform promoted progressive knowledge acquisition and reinforced behavioral intentions. Our findings align with prior work showing that gamification and biophilic design substantially enhance learner engagement and outcomes [19]. Consistent with constructivist learning theory, participants engaged in active problem-solving rather than passive reception, leading to deeper knowledge retention and attitudinal shifts [21,22]. To prevent over-reliance on extrinsic rewards, the design incorporated reflective prompts and scenario debriefings that encouraged intrinsic motivation, ethical reflection, and transfer of learning to real contexts. The Reward Marketplace thus complemented rather than replaced conceptual engagement. The observed positive orientation toward the Five Freedoms and Positive Welfare frameworks further corroborates evidence that gamified and experiential learning environments can foster not only cognitive gains but also ethical awareness [23]. Moreover, the integration of biophilic and immersive elements echoes earlier studies showing that virtual environments can elicit cognitive and emotional responses comparable to real contexts. Such environments provide robust performance-based learning experiences that support the transfer of knowledge into real-world practices [24]. Taken together, these results position biomimetic gamification as a promising approach for advancing animal welfare education, with the potential to extend beyond knowledge acquisition to cultivate motivation, ethical orientation, and practical competence. The framework is scalable across cultural and institutional contexts because its modular design allows language localization, offline functionality, and integration into existing curricula. Future iterations will test adaptability with farmers and students in diverse geographic and technological environments. Recognizing variability in technological access, the application was optimized for low-bandwidth use and mobile compatibility, with multilingual support for Arabic and English to accommodate diverse literacy levels among farmers.

Although biomimetic gamification provides an innovative bridge between ecological systems and learning design, its pedagogical impact depends on learners’ ability to perceive and interpret natural metaphors meaningfully. Future iterations will include perception studies to assess how participants relate these metaphors to real animal welfare contexts. Furthermore, implementation may face contextual challenges, including varying levels of digital literacy among farmers, cultural differences in gamification preferences, and ethical considerations in simplifying welfare scenarios into gameplay. These factors, along with the limited pilot sample, represent key boundary conditions for future validation.

## 6. Conclusions

In conclusion, this study highlights the transformative potential of biomimetic gamified learning environments in animal science education. By embedding ecological principles into gamified instructional design, GamifyWELL was designed to facilitate deeper understanding of complex biological and ethical concepts while fostering empathy and responsibility toward animal welfare. This approach not only aims to enhance engagement and knowledge retention but is also intended to promote attitudinal and behavioral shifts consistent with sustainable welfare practices. As such, biomimetic gamification offers a scalable and interdisciplinary model that bridges theory and practice, leveraging digital immersion to strengthen competencies in animal care and welfare stewardship. This study contributes to both animal welfare pedagogy and biomimetic instructional design by demonstrating how ecological principles can guide gamified learning frameworks. While initial findings indicate strong engagement, the pilot scope limits statistical generalization. Future work will include longitudinal testing and cross-cultural trials to evaluate long-term behavioral outcomes and scalability. GamifyWELL thus represents a flexible, nature-inspired platform with potential application in allied sustainability and agricultural education domains.

## Figures and Tables

**Table 1:** Sequential structure of the core learning pathway in GamifyWELL, illustrating dependent tasks and their biomimetic analogies.

Stage	Description	Biomimetic Analogy
Pre-Test (Baseline Assessment & Profiling)	Diagnostic questionnaire to assess baseline knowledge and learner profile (e.g., achievers, explorers, competitors).	Initial conditions in ecosystems; organisms start from different baseline capacities.
Level 1—Basic Awareness	Introductory stage using MCQ, image-based, and video-based questions with low difficulty.	Early-stage adaptation; exposure to basic environmental cues.
Level 2—Intermediate Practice	Same question formats, with increased complexity and application of knowledge.	Diversification of responses to varied stimuli, mirroring multisensory adaptation in nature.
Level 3—Advanced Issues	Higher-order integration of concepts, introducing the Five Freedoms framework.	Structural consolidation in ecosystems; emergence of foundational rules for survival.
Level 4—Positive Welfare	Advanced application of knowledge (play, enrichment, affiliative behaviors) at the highest difficulty level.	Transition from survival to thriving; species exhibiting play, cooperation, and enrichment behaviors.
Post-Test (Adaptation Assessment)	Final assessment of knowledge gain, attitudinal change, and behavioral intention.	Evaluation of adaptive success in ecosystems; survival and reproduction as outcomes.

**Table 2:** Enrichment pathways (independent tasks) in GamifyWELL and their biomimetic analogies.

Stage	Description	Biomimetic Analogy
Welfare Duel	Competitive engagements with peers to reinforce knowledge through rivalry.	Competitive dynamics in ecosystems; species competing for limited resources.
Your Voice for Welfare	Idea-generation tasks inviting learners to contribute novel approaches to welfare enhancement.	Niche construction and innovation within ecosystems, where organisms adapt environments through creative strategies.
Share the Welfare	Submission of photographic evidence of positive or negative welfare practices.	Social signaling and observational learning observed in group-living species.

**Table 3:** Reward Marketplace in GamifyWELL: Role-specific reinforcement mechanisms and biomimetic analogies.

User Group	Rewards	Biomimetic Analogy
Students	Digital badges, bonus points, and opportunities for scholarly co-authorship.	Developmental incentives in early-life stages; symbolic rewards fostering growth and recognition.
Farmers	Honorary plaques, structured welfare evaluations, and participation as pilot farmers in applied projects.	Keystone species in ecosystems; recognition and reinforcement tied to sustainability and resource stewardship.
Veterinary Technicians	Ambassadorial titles, certification, and opportunities to act as facilitators or trainers.	Social facilitators within animal groups; roles emphasizing cooperation, leadership, and knowledge transfer.

## Data Availability

Aggregated and non-identifiable feedback data from the pilot implementation, as well as educational materials and illustrative question formats used in the development of GamifyWELL, are available from the corresponding author upon reasonable request. No individual-level datasets were generated or analyzed beyond design validation purposes.

## Abbreviations

The following abbreviations are used in this manuscript:

MCQ	Multiple-Choice Question
PLF	Precision Livestock Farming
VR	Virtual Reality
